# First Case of Nocardia wallacei From India: A Case Report and Literature Review

**DOI:** 10.7759/cureus.53035

**Published:** 2024-01-27

**Authors:** Rahul Ranjan, Jayanthi Gunasekaran, Raunak Bir, Umesh Kumar, Rajiv M Gupta

**Affiliations:** 1 Department of Microbiology, ESIC Medical College and Hospital, Faridabad, IND

**Keywords:** nocardia spp, fatal, cotrimoxazole, amikacin, immunocompetent, immunosupressed, hiv, india, first case, nocardia wallacei

## Abstract

*Nocardia* is a type of bacteria that can cause infections in both immunocompromised and immunocompetent hosts. It is an obligate aerobe and is commonly found in the environment. Pulmonary nocardiosis may present as pneumonia, endobronchial inflammatory masses, lung abscess, and cavitary disease with contiguous extension, leading to effusion and empyema. We present a case of pulmonary nocardiosis in a 75-year-old male patient with type 2 diabetes mellitus. The patient presented with bilateral pneumonia and hypoxia with an oxygen saturation of 85%. Sputum samples were sent to the microbiology laboratory for testing. Acid-fast staining with 1% H_2_SO_4_ showed acid-fast branching filamentous rods, but *Nocardia* could not be isolated in culture. The sample was subjected to 16S rRNA gene sequencing, which identified the pathogen as *Nocardia wallacei*. The culture of the sputum did not grow any pathogenic organisms, and the blood culture was sterile. Unfortunately, the patient left the hospital against medical advice as he was advised for intubation. The patient could not survive and died the next day after leaving the hospital. *N. wallacei* can be fatal and cause disseminated infection in both immunosuppressed and immunocompetent patients. Only eight case reports of *N. wallacei *have been reported in the literature from various parts of the world. Our case is the first case report of *N. wallacei* from India.

## Introduction

*Nocardia* is an obligate aerobic, Gram-positive, and branched filamentous bacteria. It is saprophytic in nature and is commonly found in the environment [1]. Pulmonary nocardiosis can present as acute, subacute, or chronic suppurative infection with a tendency to remit or exacerbate. It can infect both immunocompromised and immunocompetent hosts. Clinically, it may present as pneumonia, endobronchial inflammatory masses, lung abscess, and cavitary disease with contiguous extension, leading to effusion and empyema [2]. *Nocardia wallacei *belongs to the *Nocardia transvalensis* complex, which is ubiquitous in the environment [3,4]. It can involve organs such as the lungs, pleura, skin and soft tissues, and brain [5]. Only a few cases of *N. wallacei* have been reported worldwide so far, which presented with few clinical features such as disseminated actinomycetoma and pulmonary abscess with dissemination to the brain [5,6]. The bacteria cannot be identified by conventional biochemical tests and require advanced techniques such as sequencing for identification [4]. All the previously published studies have confirmed the species by sequencing, so sequencing plays a major role in the identification of new or closely related species of *Nocardia* [5,6]. *N. wallacei* is known to be resistant to many drugs, making them difficult to treat [6]. Currently, there are no serological tests available for the diagnosis of active nocardiosis due to cross-reactivity among different *Nocardia* species, *Mycobacterium tuberculosis*, *Mycobacterium leprae*, and other *Actinomycetes* [4].

## Case presentation

A 75-year-old male patient was referred to our emergency department by a private physician with a provisional diagnosis of acute febrile illness with lower respiratory tract infection and bilateral pneumonia, along with severe hypoxia. The patient had been experiencing fever and shortness of breath for one month on-and-off basis, with cough and weakness. He had a known case of type 2 diabetes mellitus and epilepsy and was on treatment for the same. He was a non-smoker, non-alcoholic, and not hypertensive. There was no history of pulmonary tuberculosis, and he was a shopkeeper by profession.

On examination, the patient was conscious and oriented to time, place, and person. He was tachypneic and dyspneic, with a blood pressure of 121/86 mmHg, pulse rate of 92/minute, and respiratory rate of 25/minute. His SPO_2_ was 85% in room air. Bilateral rhonchi and crepitations were heard on auscultation of the chest, and bilateral pedal edema was also present. The patient was started on non-invasive ventilation.

**Figure 1:**
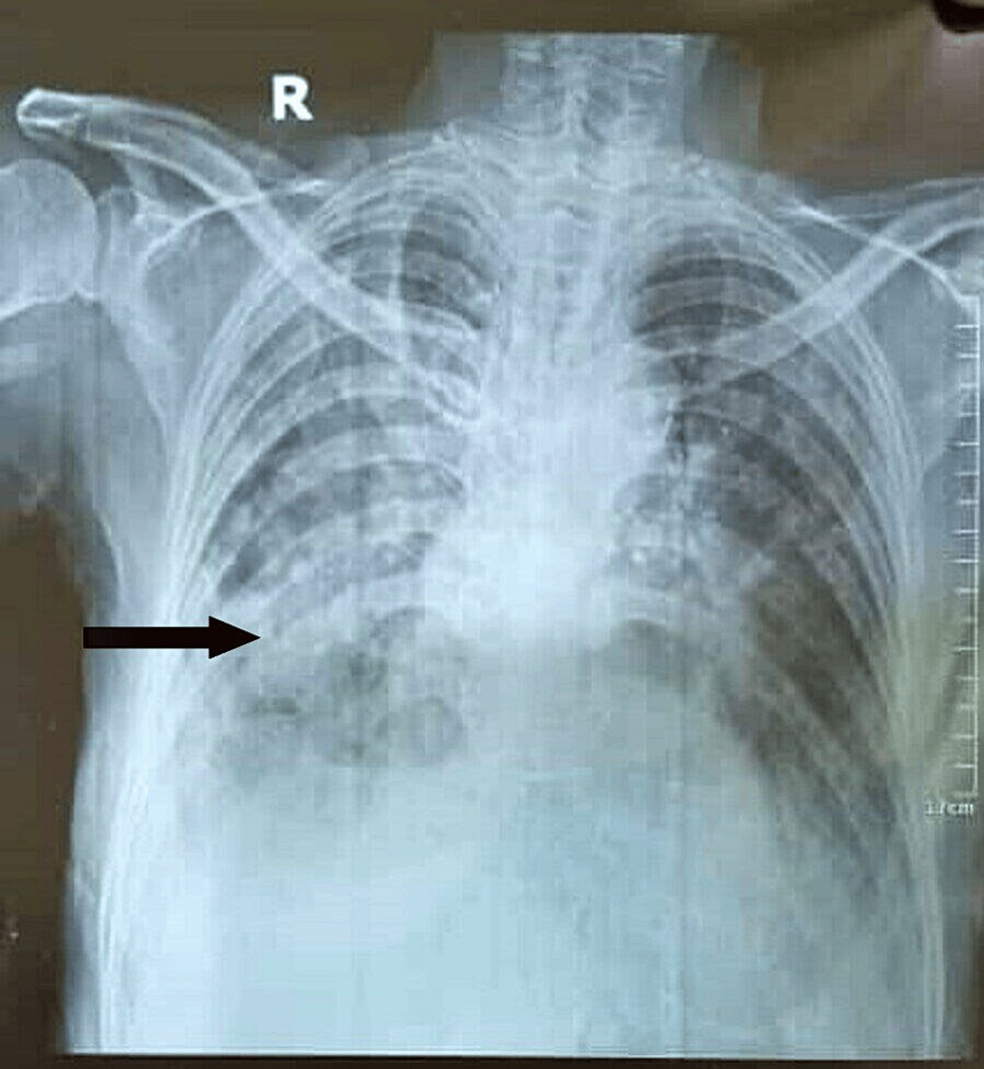
X-ray PA view chest. X-ray showing bilateral nodular opacities and infiltrates, along with areas of consolidation.

The total leucocyte count was 13,770/μL, differential leucocyte count - neutrophil (95.3%) and lymphocytes (2.1%), and hemoglobin was 10.8 g/dL. X-ray PA view showed bilateral nodular opacities and infiltrates, along with areas of consolidation (Figure 1).

**Figure 2:**
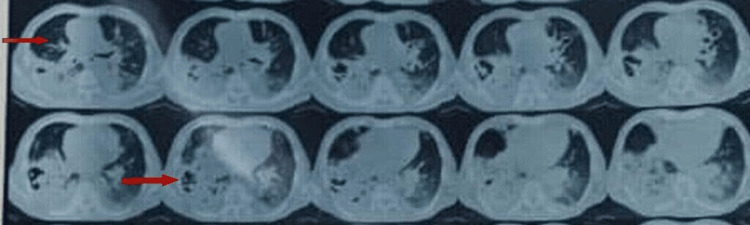
HRCT of the chest. HRCT of the chest showing multiple heterogeneously enhancing lesions of bilateral lung fields with consolidation of the right lower lobe.

HRCT of the chest shows multiple centrilobular nodules and multiple heterogeneously enhancing lesions of bilateral lung fields with consolidation of the right lower lobe (Figure 2).

Sputum and blood cultures were sent, and the patient was started empirically on IV meropenem 1 g TID and IV clarithromycin 500 mg BD. The patient’s condition was deteriorating, and the medical team planned to put him on a ventilator. However, the patient’s attenders refused and decided to take discharge against medical advice on the second day of admission.

**Figure 3:**
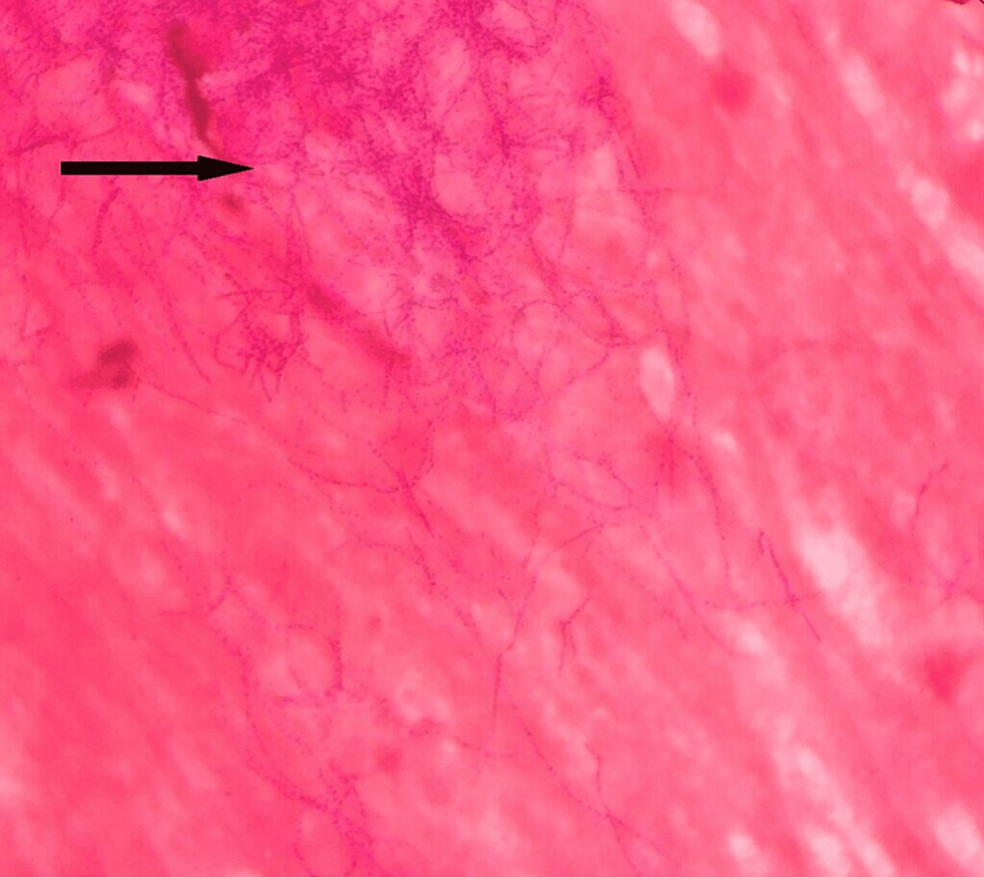
Gram stain of the sputum sample. Gram stain - showing multiple Gram-positive beaded branching filamentous rods.

Sputum samples were sent for microbiological examination, and on Gram stain, Gram-positive beaded branching filamentous bacilli were seen (Figure 3).

**Figure 4:**
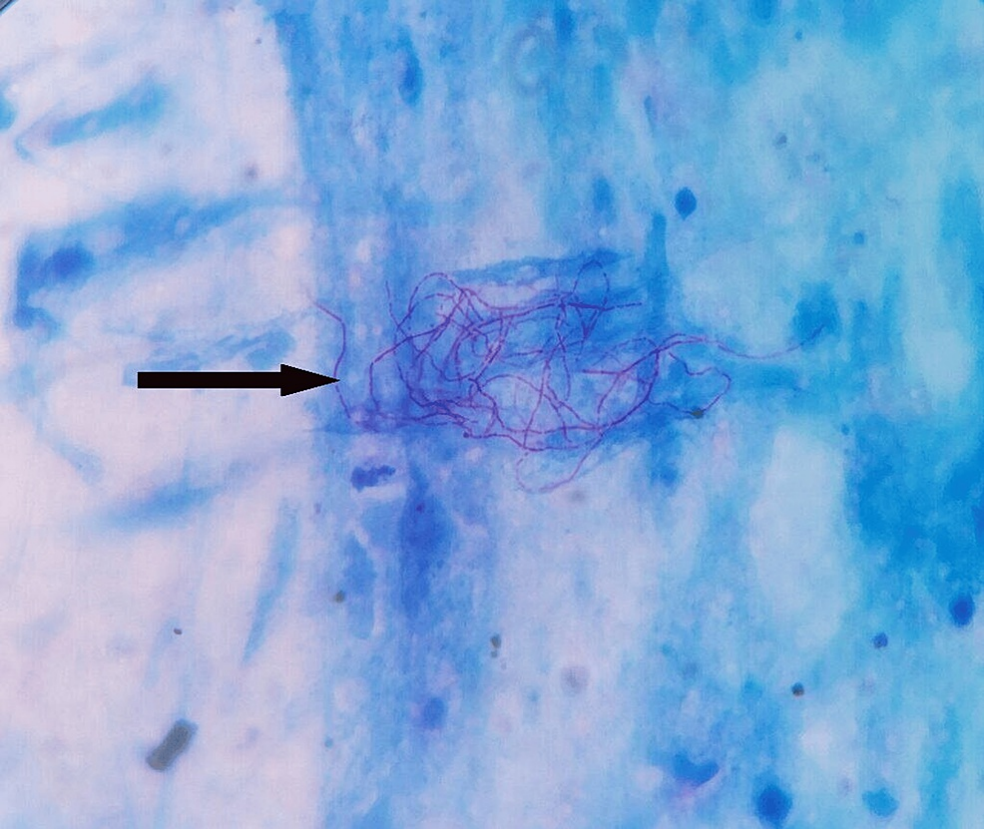
Modified acid-fast stain of sputum. Modified acid-fast stain with 1% H_2_SO_4_ showing acid-fast beaded branching filamentous rods.

The modified Ziehl-Neelsen stain (1% H_2_SO_4_) from the sample demonstrated pink-colored beaded branching acid-fast filamentous bacilli suggestive of *Nocardia* species (Figure 4).

After 48 hours of incubation, no pathogenic microorganisms could be isolated from the sputum culture. The blood culture was negative after incubation for five days. A 10% potassium hydroxide mount (KOH) showed no fungal elements. *M. tuberculosis *complex was not detected by cartridge-based nucleic acid amplification test (CBNAAT) of sputum.

In the culture of sputum, *Nocardia* could not be isolated after 72 hours of incubation of Sabouraud's dextrose agar (SDA) and blood agar. However, *Nocardia *spp. was detected by direct microscopy, and the treating doctor was informed. Unfortunately, by that time, the patient had left the hospital against medical advice. When the medical team tried to contact the patient directly, the patient’s relative informed them that the patient had died the next day after being discharged from the hospital. The sample was subjected to 16S rRNA sequencing and identified as *N. wallacei *(GenBank accession number: OR533615).

Antimicrobial susceptibility testing was not done as *Nocardia* could not be isolated in culture.

## Discussion

This is the first case report of *N. wallacei* from India. The previous cases of *N. wallacei* reported had presented with few clinical features such as disseminated actinomycetoma and pulmonary abscess with dissemination to the brain [5,6]. So far, eight case reports have been reported in the literature from various parts of the world. Of the eight cases, 50% were immunocompromised (two cases of HIV and two cases of carcinoma), and 50% were immunocompetent. Pulmonary involvement was there in 87.5% (seven out of eight) cases, and one case affected the skin and soft tissue. Four out of the seven cases (57%) of *N. wallacei *with pulmonary involvement had dissemination to the brain. Of the four cases of dissemination to the brain, 50% were immunocompromised and 50% were immunocompetent. The correlation between fatality and immune status is not conclusive as there are only a few case reports of *N. wallacei*. From the literature, it is clear that *N. wallacei* can cause both pulmonary and extrapulmonary nocardiosis and can be fatal and disseminated [7]. It is also clear that *N. wallacei* can cause disseminated infection even in immunocompetent patients [7]. It is also evident that *N. wallacei* can cause disseminated infection in both immunosuppressed and immunocompetent patients. We could clearly conclude that *N. wallacei* has a predilection to the brain and is disseminated to the brain in almost 60% of cases, although more research is required to support this evidence. Our patient was a known diabetic and elderly, which could be the reason for immunosuppression and lead to fatality. Therefore, diagnosing and treating *N. wallacei*-infected patients early can save the patient’s life. There are only a few cases discussed in the literature, and a short summary of all the cases is discussed in Table 1.

**Table 1:** Summary of Nocardia wallacei cases reported in the past.

Study, year	Place	Age/Sex	Associated Risk Factors/Underlying Disease	Site of infection	Clinical presentation	Identified based on	Treatment given	Outcome
Present case, 2023	Faridabad, India	75 years/Male	Diabetes mellitus and Elderly	Lungs	Fever, cough and difficulty breathing for 1 month	16s rRNA sequencing	Patient left the hospital against medical advice	Died
Quin et al. [5], 2023	China	59 years/Female	Immunocompetent	Lungs and subcutaneous	Breathing difficulty and Lump in the back (coinfection of mycobacterium abscesses and Nocardia wallacei)	Next generation sequencing	Cotrimoxazole + doxycycline, clarithromycin and cefoxitin	Patient recovered
Palomba et al. [6], 2022	Milan, Italy	80 years/Male	Known case of lymphoblastic lymphoma on treatment	Brain, disseminated from lungs	Disorientation and memory deficiency for 1 month	16s rRNA sequencing	Cotrimoxazole and linezolid	Patient recovered
Sithamraju et al. [7], 2020	Texas, United States	46 years/Male	Immunocompetent	Lungs and brain	Disseminated multiple abscesses in the lungs and brain	16sr RNA sequencing	Cotrimoxazole, amikacin and imipenem- cilastatin. Duration not mentioned	Not Mentioned
Welsh et al. [8], 2018	Monterrey, Mexico	18 years/Female	Immunocompetent	Skin and soft tissue	Disseminated actinomycetoma	Not Mentioned	Linezolid	Patient recovered
González-Nava et al. [9], 2016	Mexico	43 years/Female	HIV patient	Lungs	Breathing difficulty and productive cough	16s rRNA sequencing	Not mentioned	Not Mentioned
Cassir et al. [10], 2013	Marseille, France	62 years/Female	Immunocompetent	Lungs and disseminated to the brain	Fever, cough and chest pain and increasing headache	16s rRNA sequencing	Cotrimoxazole and linezolid	Patient recovered
Hamid et al. [11], 2013	Saudi Arabia	54 years/Male	HIV patient	Lungs	Fever, cough and difficulty breathing for a long time	16s rRNA sequencing	Not mentioned	Patient recovered
Conville et al. [12], 2008	Texas, United States	57 years/Male	Known case of oral carcinoma	Pleura and disseminated to the brain	Fever, cough and chest pain for 3 weeks	16s rRNA sequencing	Cotrimoxazole and amikacin	Died

## Conclusions

*N. wallacei* pulmonary cases had shown a predilection of dissemination to the brain in 60%. It is also known to be multidrug-resistant, including drugs such as amikacin and clarithromycin. *N. wallacei* can cause dissemination equally in immunocompetent and immunocompromised patients. Therefore, irrespective of the immune status of the patient, diagnosing *N. wallacei* and starting treatment as early as possible can be life-saving. To better understand the range of symptoms, spread, and mortality of *N. wallacei*, more case reports from different regions of the world are needed.
